# GABA Receptor Agonists Protect From Excitotoxic Damage Induced by AMPA in Oligodendrocytes

**DOI:** 10.3389/fphar.2022.897056

**Published:** 2022-07-26

**Authors:** Laura Bayón-Cordero, Blanca Isabel Ochoa-Bueno, Asier Ruiz, Marina Ozalla, Carlos Matute, María Victoria Sánchez-Gómez

**Affiliations:** ^1^ Laboratory of Neurobiology, Achucarro Basque Center for Neuroscience, Leioa, Spain; ^2^ Department of Neurosciences, University of the Basque Country (UPV/EHU), Leioa, Spain; ^3^ Centro de Investigación Biomédica en Red de Enfermedades Neurodegenerativas (CIBERNED), Leioa, Spain

**Keywords:** GABA receptor, oligodendrocyte, AMPA, baclofen, muscimol, excitotoxicity, multiple sclerosis

## Abstract

Oligodendrocytes are the myelin forming cells of the central nervous system, and their vulnerability to excitotoxicity induced by glutamate contributes to the pathogenesis of neurological disorders including brain ischemia and neurodegenerative diseases, such as multiple sclerosis. In addition to glutamate receptors, oligodendrocytes express GABA receptors (GABAR) that are involved in their survival and differentiation. The interactions between glutamate and GABAergic systems are well documented in neurons, under both physiological and pathological conditions, but this potential crosstalk in oligodendrocytes has not been studied in depth. Here, we evaluated the protective effect of GABAR agonists, baclofen (GABA_B_) and muscimol (GABA_A_), against AMPA-induced excitotoxicity in cultured rat oligodendrocytes. First, we observed that both baclofen and muscimol reduced cell death and caspase-3 activation after AMPA insult, proving their oligoprotective potential. Interestingly, analysis of the cell-surface expression of calcium-impermeable GluR2 subunits in oligodendrocytes revealed that GABAergic agonists significantly reverted GluR2 internalization induced by AMPA. We determined that baclofen and muscimol also impaired AMPA-induced intracellular calcium increase and subsequent mitochondrial membrane potential alteration, ROS generation, and calpain activation. However, AMPA-triggered activation of Src, Akt, JNK and CREB was not affected by baclofen or muscimol. Overall, our results suggest that GABAR activation initiates alternative molecular mechanisms that attenuate AMPA-mediated apoptotic excitotoxicity in oligodendrocytes by interfering with expression of GluR subunits in membranes and with calcium-dependent intracellular signaling pathways. Together, these findings provide evidence of GABAR agonists as potential oligodendroglial protectants in central nervous system disorders.

## Introduction

Oligodendrocytes are the myelin forming cells in the central nervous system (CNS), and they express multiple neurotransmitter receptors, including glutamate receptors (GluR) (Yoshioka et al., 1995; Matute et al., 1997; McDonald et al., 1998). Excitotoxic damage induced in oligodendrocytes by overactivation of glutamate receptors leads to oligodendrocyte death, and it is a contributor to the pathogenesis of CNS-related disorders including ischemia, traumatic brain injury, and neurodegenerative diseases such as multiple sclerosis, where oligodendrocyte death is a well-known pathological hallmark (Matute et al., 2001; Matute et al., 2006; Matute et al., 2007). Excitotoxicity is associated with sustained activation of glutamate ionotropic receptors, in particular α-amino-3-hydroxy-5-methyl-4-isoxazolepropionate (AMPA) and kainate receptors, sensitive to activation with these agonists. In oligodendrocytes, AMPA-activated GluR receptors are mainly formed by GluR1-4 subunits (Matute et al., 2007).

Excitotoxic insults to oligodendrocytes are dependent on calcium entry through ionotropic GluRs, which alters calcium homeostasis, induces changes in mitochondrial function and activates apoptotic pathways involving caspases-9 and -3, leading to oligodendroglial cell death (Galluzzi et al., 2009; Sánchez-Gómez et al., 2011; Simonishvili et al., 2013). In these excitotoxic processes, calcium is an essential signaling molecule that affects pivotal cellular mechanisms. The increase in cytosolic calcium levels directly targets the mitochondria, leading to an alteration in the polarization of the mitochondrial membrane (Duchen, 2000) and causing overproduction of reactive oxygen species (ROS) and reduced cell survival (Sánchez-Gómez et al., 2003; Ness et al., 2004; Suski et al., 2018; Singh et al., 2019). Alterations in calcium levels also affect calpain activity. Calpains are calcium dependent cysteine proteases that are ubiquitously expressed as two isoforms, μ- and m-calpain, which are activated by micromolar and millimolar concentrations of calcium, respectively. The potential role of calpains in cell death is indicated by a growing list of substrates, including Bax, p53, PARP, Src, Akt, JNK, and CREB, whose proteolytic cleavage activity has been characterized as crucial in oligodendrocyte excitotoxicity, acute hypoxia, traumatic brain injury, and chronic degeneration (Trinchese et al., 2008; Sánchez-Gómez et al., 2011; Barateiro et al., 2012; Hossain et al., 2013; Wang et al., 2013; Jantzie et al., 2016; Zhang et al., 2017).

While glutamate is the main excitatory neurotransmitter in the CNS, γ-aminobutyric acid (GABA) is the major inhibitory neurotransmitter. Excitatory-inhibitory signal balance is necessary to ensure proper functioning of cells, therefore, correct crosstalk between glutamate and GABAergic signaling is essential (Kantamneni, 2015). Oligodendrocyte progenitor cells receive both excitatory inputs mediated by glutamate and inhibitory signals mediated by GABA (Lin and Bergles, 2004; Káradóttir et al., 2008; Kukley et al., 2008), which supports the importance of these neurotransmitters in the fate and function of the oligodendroglial lineage. Along this line, it has been described that increasing GABAergic action can prevent excitotoxicity and oligodendrocyte loss following preterm birth by creating a normal balance of inhibition-excitation (Shaw et al., 2021).

Disturbances in GABAergic signaling are found in several injury conditions, such as stroke or epilepsy (Bai et al., 2021), which gives insight into their potential relevance in the progression of these disorders. Oligodendrocytes express the two main GABA receptors (GABAR), ionotropic GABA_A_R and metabotropic GABA_B_R (Serrano-Regal et al., 2020), and the relevance of GABARs for oligodendrocyte functionality has become clear in recent years (reviewed in Serrano-Regal et al., 2020; Bai et al., 2021). GABARs are related to myelination and neuroprotection in the CNS, given the link between GABA_A_R signaling disruption or downregulation and reduced myelination observed *in vivo* (Zonouzi et al., 2015; Kalakh and Mouihate, 2019), or the decreased myelination following GABA_A_R activation observed in organotypic slices (Hamilton et al., 2017), and considering the remyelinating capacity of GABA_B_R agonist baclofen following spinal cord injury (Serrano-Regal et al., 2022).

Here, we investigated the impact of GABA_A_ and GABA_B_R agonists, muscimol and baclofen, respectively, in AMPA-mediated excitotoxicity using primary cultures of cortical rat brain-derived oligodendrocytes, mimicking the excitotoxic insults through moderate activation of AMPA receptors. Our results provide evidence of the protective effect of baclofen and muscimol from AMPA-induced excitotoxic death of oligodendrocytes through modulation of cell-surface GluR2 AMPA subunit expression in these cells, as well as by regulating the subsequent cytosolic calcium overload calpain activation and mitochondrial dysfunction.

## Materials and Methods

### Animal Ethic Statement

The animal study was approved by the internal Animal Ethics Committee of the University of the Basque Country (UPV/EHU) and the European Communities Council Directive. All efforts were made to minimize animal suffering and the number of animals used. Sprague-Dawley rats of both sexes were used for the experiments.

### Rat Brain Oligodendrocyte Primary Culture and Excitotoxicity Induction

Oligodendrocyte progenitor cell (OPC) culture was obtained from newborn Sprague-Dawley rat mixed glial cultures as previously described (Sánchez-Gómez et al., 2018). Isolated OPCs were seeded onto poly-D-lysine-coated coverslips and cultured at 37°C with 5% CO_2_ in SATO differentiation medium for 2–3 days, to promote maturation into oligodendrocytes (Canedo-Antelo et al., 2018). Excitotoxic conditions were recreated using oligodendrocytes by exposure to cyclothiazide (CTZ; 100 μM; Tocris) for 10 min before incubation with AMPA (10 μM; Tocris) for 30 min (Sánchez-Gómez and Matute, 1999; Sánchez-Gómez et al., 2011). After incubation with AMPA, the medium was changed to remove the stimulus and for treatments with baclofen or muscimol; these GABAergic drugs were added again after AMPA removal to maintain their effect until the end of the experiment.

### Cell Viability Assay

Cultured oligodendrocytes were exposed to excitotoxicity and 24 h later, they were loaded with calcein-AM (1 μM; Invitrogen) for 30 min at 37°C. Fluorescence was measured in a Synergy H4 hybrid reader fluorimeter (Bio-Tek Instruments), with excitation at 485 nm and emission at 528 nm.

### Western Blotting

Isolated oligodendrocytes were scraped into RIPA buffer supplemented with EDTA and protease inhibitor cocktail (Thermo Scientific). Samples were diluted in sodium dodecyl sulfate sample buffer and boiled for 8 min at 100°C. Protein extracts were separated by size through SDS-PAGE in 4%–20% Criterion TGX precast gels and then transferred to Trans-Blot Turbo Midi Nitrocellulose or PVDF Transfer Packs (Bio-Rad). Membranes were blocked with 5% BSA (Sigma-Aldrich) or phosphoBLOCKER reagent (Cell Biolabs) and incubated with the following primary antibodies: rabbit anti-pSrc (#2101), anti-Src (#2109), anti-pAkt (#9271), anti-Akt (#9272), anti-pJNK (#9251), anti-JNK (#9252), anti-pCREB (#9198) (1:1000; all from Cell Signaling) and mouse anti-pJNK (#sc-6254), anti-JNK (#sc-7345) (1:500; both from Santa Cruz), anti-CREB (#9104) and anti-GAPDH (#mab374; Merck). Horseradish peroxidase-conjugated goat anti-rabbit or sheep anti-mouse (1:2000; Cell Signaling) were used as secondary antibodies. Protein band signals were developed using SuperSignal West Femto chemiluminescent substrate detection kit (Thermo Scientific) and images were acquired with a ChemiDoc MP image system (Bio-Rad). For incubation with primary antibodies against the total portion of the protein, antibodies against phosphorylated proteins were stripped by incubation in Restore Western Blot stripping buffer (Thermo Scientific). Ponceau Red staining, GAPDH, or the total portion of the protein was used for normalization of the signal.

### Immunofluorescence and Image Analysis

Oligodendrocytes were fixed in 4% paraformaldehyde for 20 min at RT. For cleaved caspase-3 detection, membranes were permeabilized in blocking solution containing 0.1% Triton X-100 and 4% normal goat serum in PBS and then labeled with rabbit anti-cleaved caspase-3 (1:500; Cell Signaling; #9661) and mouse IgM anti-O4 (1:100; R&D Systems; #MAB1326) overnight at 4°C. Then, cells were incubated with goat anti-rabbit IgG Alexa Fluor-488 (1:500; Invitrogen; #A11034), goat anti-mouse IgM TXRed (1:500; Thermo Scientific, #401296). For detection of the N-terminal extracellular domains of GluR2 and GABA_B1_R, cells were blocked using 2% normal goat serum. Primary antibodies, mouse anti-GluR2 extracellular (1:1500; Merck, #MAB397) and rabbit anti-GABA_B1_ extracellular (1:200; Alomone, #AGB-001) were added for 1 h at 37°C, after which the cells were incubated with goat anti-mouse IgG Alexa Fluor-488 and goat anti-rabbit IgG Alexa Fluor-594 (1:500; Invitrogen; #A11001 and ##A11012 respectively). 4′,6-diamidino-2-phenylindole (DAPI; 4 μg/ml; Sigma-Aldrich) was used for nuclear staining and coverslips were mounted using ProLong™ Gold anti-fade reagent (Invitrogen).

Images were acquired using a 40X oil-immersion objective (numerical aperture 1.3) on an inverted Zeiss LSM800 confocal microscope (Analytical and High Resolution Microscopy Service in Biomedicine, UPV/EHU) for cleaved caspase-3 analysis or a Leica TCS STED CW SP8 confocal microscope (Achucarro Basque Center for Neuroscience) for receptor expression analysis. Image analysis was performed using ImageJ software (National Institute of Health). For receptor expression quantification, individual cells were selected as regions of interest (ROIs) and the integrated density value was measured for each ROI. In this case, ten fields of view were quantified per biological replicate. For cleaved caspase-3 quantification, seven fields of view were quantified per biological replicate.

### Cytosolic Calcium Imaging

Cytosolic calcium levels in oligodendocytes were measured as described previously (Ruiz et al., 2014). Briefly, cells were incubated with Fluo4-AM (1 mM; Molecular Probes, Invitrogen) for 30 min at 37°C, and then exposed to AMPA (10 μM) plus CTZ (100 μM). Fluorescence was imaged through a 40X objective (numerical aperture 1.3) on an inverted Zeiss LSM800 confocal microscope (Analytical and High Resolution Microscopy Service in Biomedicine, UPV/EHU) at an acquisition rate of 1 frame/15 s for 5 min. For data analysis, a population of 15–25 cells per coverslip was selected and the oligodendrocyte soma was selected as ROI. Calcium levels are expressed as F/F0 ± SEM (%), in which F represents the fluorescence value for a given time point and F0 represents the mean of the resting fluorescence level. Background values were subtracted in all cases. The area under the curve of accumulated calcium levels (Fluo4-AM fluorescence increase) was calculated during the time course.

### Mitochondrial Membrane Potential Gradient Measurement

Oligodendrocytes were exposed to excitotoxicity and loaded with 5,5′,6,6′-tetrachloro-1,1′,3,3′-tetraethylbenzimidazolcarbocyanine iodide (JC-1; 3 μM; Invitrogen) for 15 min at 37°C. After changing medium, the coverslips were washed with HBSS without phenol red and transferred to a different plate. Fluorescence was monitored every 15 min for 2 h using a Synergy H4 hybrid reader fluorimeter (Bio-Tek Instruments), with excitation at 485 nm and emission at 528 nm for green (monomeric form, cytosol) and at 620 nm for red fluorescence (aggregated form, mitochondrial matrix). Changes in the mitochondrial potential gradient are indicated by the red/green ratio.

### Measurement of Intracellular Reactive Oxygen Species

Cells were loaded with 5-(and 6)-chloromethyl-2 ´,7-dichlorodihydro fluorescein diacetate acetyl ester (CM-H2DCFDA; 10 μM; Invitrogen) for 30 min at 37°C. Fluorescence was measured using a Synergy H4 hybrid reader fluorimeter (Bio-Tek Instruments), with excitation and emission at 485 and 528 nm, respectively. ROS production values were normalized using the calcein-AM probe (1 μM) in duplicate wells seeded under the same conditions.

### Calpain Activity Assay

Calpain activity was measured in oligodendrocytes using the Calpain-Glo protease assay (Promega) and luminescence was monitored after excitotoxicity exposure every 15 min for 90 min using a Synergy H4 hybrid reader fluorimeter-luminometer (Bio-Tek Instruments).

### Statistical Analysis

Statistical analysis was performed using GraphPad Prism software version 8.0. All data are expressed as the mean ± SEM. The number of biological replicates per experiment is indicated in each case and experiments were performed at least twice. For comparisons between multiple experimental groups, one-way analysis of variance (ANOVA) with Fisher’s LSD test was applied, unpaired *t* test was used for comparisons between two experimental groups, and *p*<0.05 was considered significant.

## Results

### Baclofen and Muscimol Attenuate Excitotoxic Cell Death in Oligodendrocytes

**FIGURE 1:**
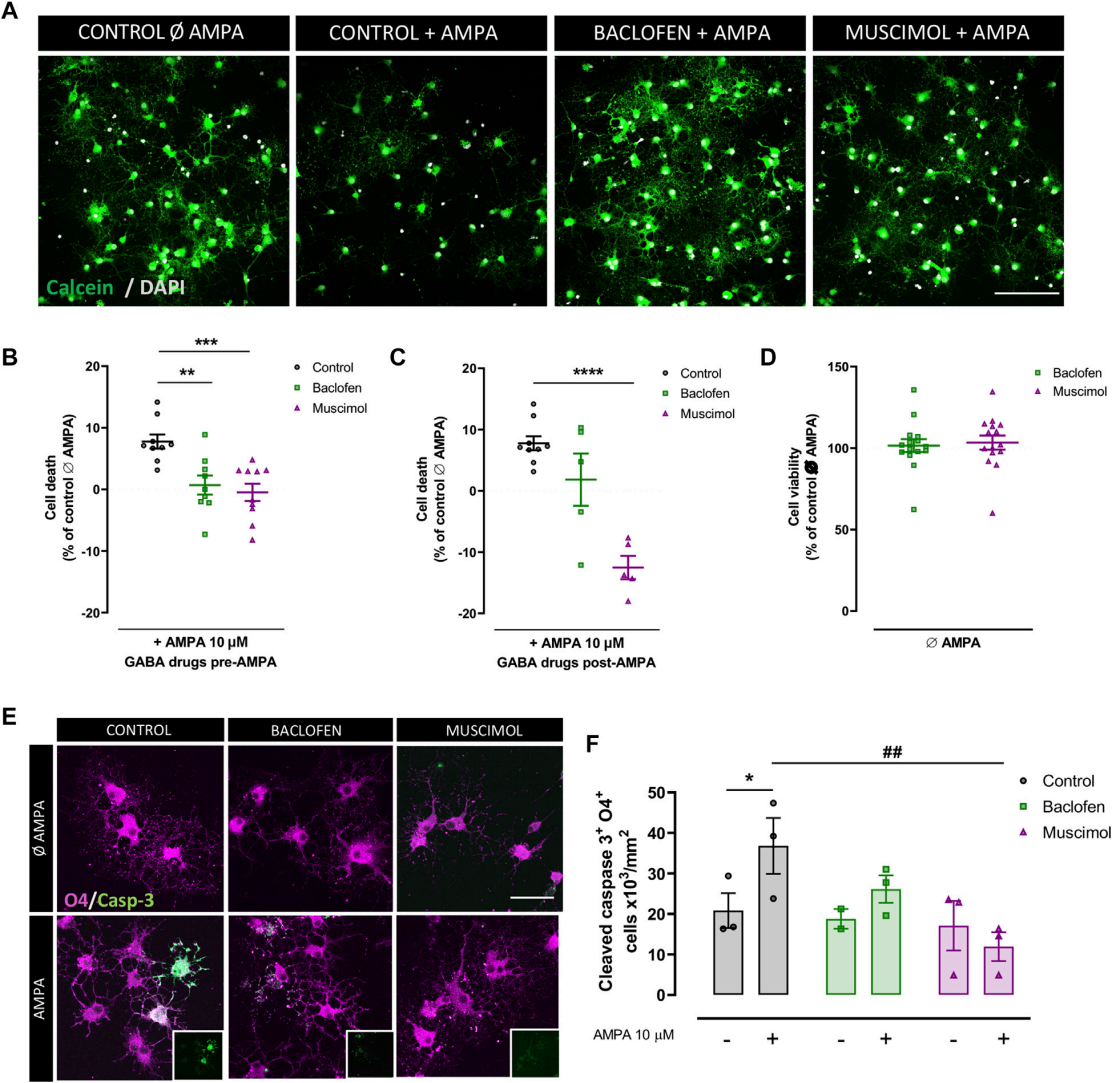
**Baclofen and muscimol reduce cell death AMPA‐induced excitotoxicity in oligodendrocytes**. **(A)** Representative images from viability assays of oligodendrocytes treated with CTZ and AMPA plus baclofen or muscimol added before the excitotoxic insult. Cells are labeled with calcein (green) and DAPI (blue). Scale bar: 100 µm. **(B, C)** Percentage of death oligodendrocytes after 24h in cultures treated with baclofen or muscimol 30 min before or after exposure to CTZ and AMPA, respectively. All conditions were normalized to calcein values from untreated cells, which was considered as 0% of cell death. **(D)** No cell death was observed in oligodendrocytes treated with baclofen or muscimol and analyzed 24 h later. All conditions were normalized to calcein values from untreated cells, which was considered as 100% of cell viability. Data are shown as mean ± SEM of at least 5 independent experiments. **p<0.01, ***p<0.001, ****p<0.0001 vs. control, one‐way ANOVA. **(E)** Representative images of oligodendrocytes treated with CTZ and AMPA plus baclofen or muscimol added before the excitotoxic insult, and labeled with rabbit anti‐cleaved caspase 3 (green) and IgG anti‐O4 (magenta). Scale bar: 50 µm. **(F)** Number of cleaved caspase 3‐ and O4‐positive cells per area. Data are shown as mean ± SEM of at least 2 independent experiments. *p<0.05 vs. control Ø AMPA; ##p<0.01 vs. control; one‐way ANOVA.

To assess the protective effect of the GAB_A_R agonist, muscimol, and the GABA_B_R agonist, baclofen, in oligodendrocyte excitotoxicity, we first mimicked the excitotoxic signal in vitro through administration of CTZ and AMPA (we will refer to AMPA addition as the excitotoxic signal), and we analyzed cell viability using the calcein-AM probe (Figures 1A–D). When baclofen and muscimol were added 30 min before AMPA and maintained after AMPA stimulus (pre-AMPA), both drugs reduced the percentage of cell death caused by AMPA (Figure 1B), while muscimol alone effectively attenuated excitotoxic cell death when added only after AMPA (post-AMPA) (Figure 1C). Moreover, neither baclofen nor muscimol caused any variation in cell viability in the absence of AMPA (Figure 1D), showing that neither of these GABAR agonists presented toxicity against oligodendrocytes in vitro. The most reproducible protective effect was observed when drugs were added 30 min before and maintained during AMPA stimulus, thus, further analyses were mainly performed following this drug treatment protocol.

Given the link between AMPAR overactivation and apoptosis activation (Sánchez-Gómez et al., 2003, Sánchez-Gómez et al.,2011; Canedo-Antelo et al., 2018), we next evaluated whether baclofen and muscimol reduced the expression of cleaved caspase-3 in O4-positive oligodendrocytes (Figures 1E,F). We verified that the AMPA-induced increase in cleaved caspase-3 was not significantly altered by baclofen addition, but was attenuated by muscimol treatment (Figure 1F). Thus, selective activation of GABAR in oligodendrocytes with baclofen or muscimol protected them from excitotoxic damage induced by AMPA; muscimol seemed to be primarily responsible for reducing the caspase-dependent apoptotic pathway.

### Baclofen and Muscimol Reduce GluR2 Subunit Internalization Induced by AMPA in Oligodendrocytes

**FIGURE 2:**
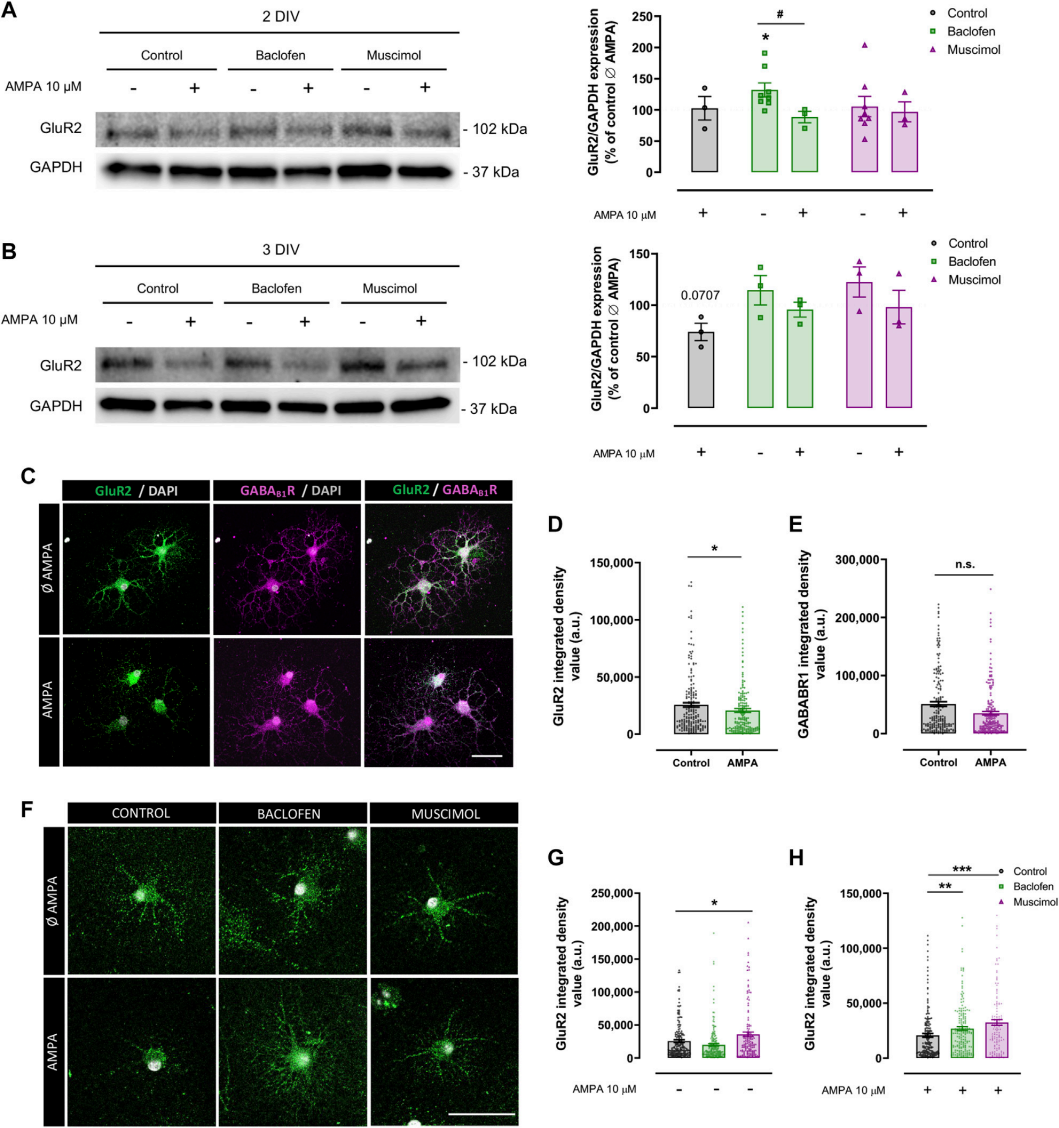
**Baclofen and muscimol reduce downregulation of cell‐surface GluR2 subunit levels induced by AMPA in oligodendrocytes**. **(A)** Western blot of GluR2 total protein levels normalized to GAPDH in oligodendrocytes treated daily with baclofen or muscimol and analyzed at day 2 in vitro (2DIV), 24 h after exposure to AMPA. **(B)** Western blot of GluR2 total protein levels in oligodendrocytes treated daily with baclofen or muscimol and analyzed at day 3 in vitro (3DIV), 24 h after exposure to AMPA. In both A and B, all conditions were normalized to control values from non‐treated cultures. Data are shown as the mean ± SEM of at least 3 independent experiments. *p<0.05 vs. control Ø AMPA; #p<0.05 vs. control; one‐way ANOVA. **(C, F)** Representative images of sum representation **(C)** or single stack **(F)** of oligodendrocytes treated or not with AMPA and/or baclofen or muscimol and stained with mouse anti‐GluR2 extracellular (green) and/or rabbit anti‐ GABA_B_R1 extracellular (magenta) and DAPI (grey). Scale bar: 50 µm. **(D, E)** Integrated density values of GluR2 or GABAB_R_1 fluorescence signal, respectively. **(G, H)** Integrated density values of GluR2 in oligodendrocytes under different treatments. Data are shown as the mean ± SEM of at least 123 individual cells. *p<0.05, **p<0.01, ***p<0.001 vs. control without or with AMPA respectively; one‐way ANOVA for multiple experimental groups comparisons; unpaired t test for two experimental group comparison.

Next, we explored the mechanisms through which baclofen and muscimol could be exerting their protective effect against AMPA-induced damage in oligodendrocytes. Taking into account the increasing evidence that GABARs play an important role in modulating the expression and function of GluRs (Kantamneni, 2015; Shaw et al., 2021), we initially assessed whether GABAergic drugs caused alterations in the expression of GluRs in oligodendrocytes under excitotoxicity insults. We focused on the Ca^2+^-impermeable GluR2 subunit, which is expressed in oligodendrocytes and whose cell-surface expression presents differential levels in response to AMPAR activation (Hossain et al., 2014; Harlow et al., 2015). To evaluate that response, AMPA stimulus was added to oligodendrocytes at 1 or 2DIV, and sample collection was performed 24 h later, at 2 or 3DIV (Figure 2). In these experiments, baclofen or muscimol was added daily until AMPA stimulus, and maintained after its removal. Protein expression analysis by immunoblot of the total protein fraction at 2DIV did not reveal any differences in the expression of GluR2 in cells treated with AMPA compared with control; baclofen or muscimol did not modify this situation (Figure 2A). Interestingly, GluR2 expression at 2DIV was higher following baclofen treatment, but this increase was reversed when baclofen addition was combined with AMPA. At 3DIV, we observed a non-significant decrease in the total expression of GluR2 in cells treated with AMPA (Figure 2B).

Considering that these analyses had been performed with total protein extracts, we proposed to specifically assess the GluR2 subunit located on the extracellular side of the membrane in oligodendrocytes at 3DIV by immunofluorescence assay (Figures 2C,F). We found that AMPA provoked a significant decrease in extracellular GluR2 level, indicating that AMPA stimulation caused internalization of GluR2 (Figures 2C,D). In parallel, we did not detect significant alterations in the density of extracellular GABA_B1_R in AMPA-treated oligodendrocytes compared to control cells (Figures 2C,E). We then evaluated the impact of baclofen or muscimol treatments on extracellular GluR2 expression and observed that, in the absence of AMPA, muscimol but not baclofen led to higher levels of cell-surface GluR2 expression (Figures 2F,G). However, in the presence of the excitotoxic effect, both drugs reduced the downregulation of extracellular GluR2 subunits induced by AMPA, although the effect caused by muscimol was more robust than baclofen (Figures 2F,H). These results suggest that baclofen and muscimol maintain extracellular membrane GluR2 levels in oligodendrocytes subjected to AMPA, which could give them greater resistance to the excitotoxic stimulus.

### Baclofen and Muscimol Compromise Calcium Signaling Caused by Excitotoxicity in Oligodendrocytes

**FIGURE 3:**
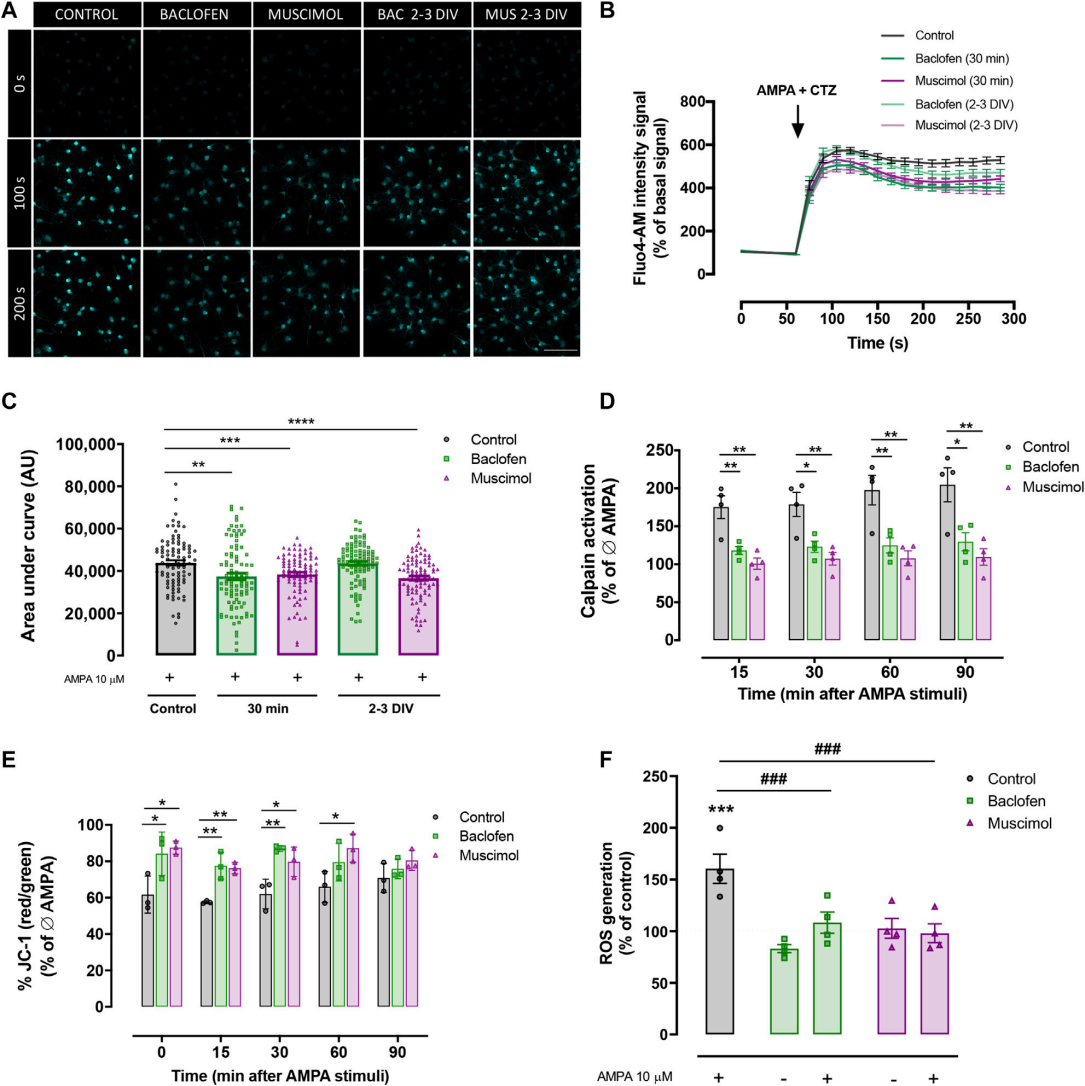
**Baclofen and muscimol modulate AMPA‐induced calcium signaling and mitochondrial alterations**. **(A)** Representative images of Ca2+ responses to CTZ and AMPA in cells loaded with Fluo4‐AM at 0 s, 100 s and 200 s of recording. **(B)** Recordings of Ca2+ responses to CTZ and AMPA in control oligodendrocytes (black trace), treated with baclofen or muscimol for 30 min before exposure to AMPA (green and magenta traces respectively, bold) or treated daily with baclofen or muscimol until the analysis (green and magenta traces respectively, soft). **(C)** Area under the curve of calcium recordings for each condition. Data are shown as violin plots indicating the median and quartiles of at least 88 cells from 4 independent experiments. **p<0.01, ***p<0.001, ****p<0.0001 vs. control; one‐way ANOVA. **(D)** Calpain activity detected using the Calpain‐Glo Protease Assay in oligodendrocytes treated with baclofen or muscimol 30 min pre‐AMPA and analyzed 30 min after CTZ and AMPA stimulus. All conditions were normalized to controls without AMPA treatment. Data are shown as the mean mean ± SEM of 4 independent experiments. *p<0.05, **p<0.01 vs. control; one‐way ANOVA. **(E)** Mitochondrial membrane potential monitored in oligodendrocytes loaded with JC‐1 probe. All conditions were normalized to controls without AMPA treatment. Data are shown as the mean ± SEM of 3 independent experiments. *p<0.05, **p<0‐01 vs. control; one‐way ANOVA. **(F)** Intracellular levels of ROS in oligodendrocytes treated with baclofen or muscimol for 30 min before adding AMPA and analyzed 30 min after AMPA stimulus. Cell were loaded with CM‐H2DCFDA probe and the values were normalized with calcein signal. All conditions were normalized to control values in cultures without AMPA treatment. Data are shown as the mean ± SEM of 4 independent experiments. ***p<0.001 vs. control Ø AMPA; ###p<0.001 vs. control; one‐way ANOVA.

Increased calcium uptake is a common feature of excitotoxic damage in oligodendrocytes, which leads to a series of events resulting in cell stress and eventually cell death (Sánchez-Gómez and Matute 1999). Considering this, we monitored changes in intracellular calcium levels in response to AMPA stimulus, to determine whether baclofen or muscimol were able to reduce calcium uptake in response to AMPA. We induced an acute AMPA stimulus during cell recording and combined it with baclofen or muscimol added daily until the day of recording (2-3 DIV), or added 30 min before recording (Figures 3A–C). Representative measurements from live imaging showed that AMPA notably increased the fluorescence signal that positively correlated with intracellular calcium concentration (Figure 3A). We observed that oligodendrocyte incubation with baclofen or muscimol 30 min before AMPA reduced the magnitude of calcium uptake driven by AMPA, while treatment during 2-3 DIV with these drugs resulted in muscimol but not baclofen reducing the calcium response (Figures 3B,C). These data indicate that baclofen and muscimol reduce calcium influx induced by AMPA-mediated GluR activation in oligodendrocytes, after both chronic and acute pretreatment.

Among the damaging consequences of disruption of calcium homeostasis due to excitotoxic insults is the enhancement of calcium-dependent calpain protease activity, leading to an activation of apoptotic pathways (Sánchez-Gómez et al., 2011; Zhang et al., 2017). With the aim of elucidating whether baclofen or muscimol affected AMPA-induced calpain activity, we used the Calpain-Glo protease assay and determined calpain activation from 15 to 90 min after AMPA, in the absence or presence of GABAergic agonists 30 min before (Figure 3D). Our results showed that AMPA-induced calpain activation was reduced when combined with baclofen or muscimol at all analyzed time points. In addition, we evaluated the mitochondrial parameters that are altered in excitotoxicity because of calcium influx, such as mitochondrial membrane potential and ROS generation. First, the JC-1 probe was used to assess changes in the mitochondrial membrane potential gradient in oligodendrocytes exposed to AMPA in the presence or absence of GABAergic drugs, and the analysis was performed from 0 to 90 min after AMPA (Figure 3E). We observed that the presence of either baclofen or muscimol were able to prevent the reduction of mitochondrial membrane potential caused by AMPA, from 0 to 60 min after AMPA stimulus. ROS generation was measured using the CM-H2DCFDA probe and the analysis revealed how baclofen and muscimol prevented the increase in ROS levels caused by AMPA incubation (Figure 3F). Taken together, these results indicate that baclofen and muscimol can prevent AMPA-triggered calcium signaling in oligodendrocytes through calpain activation and mitochondrial dysfunction.

### Baclofen and Muscimol Do Not Interfere With Src, Akt, JNK, or CREB Activated by AMPA in Oligodendrocytes

**FIGURE 4:**
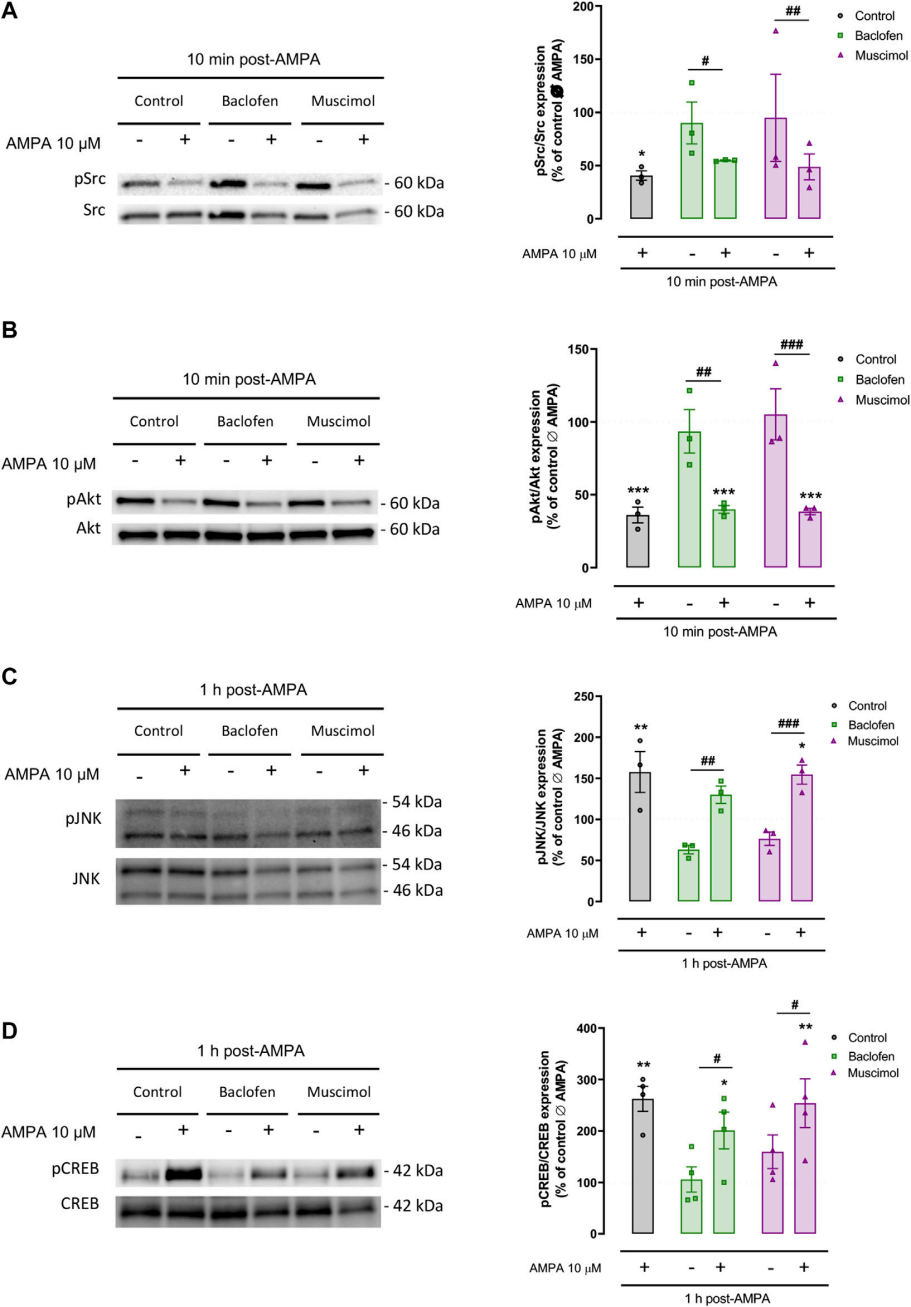
**Baclofen and muscimol do not modulate AMPA‐activated Src, Akt, JNK and CREB signaling pathways**. Western blot of phosphorylated Src **(A)**, Akt **(B)**, JNK **(C)** and CREB **(D)** protein levels, normalized to the total protein, in oligodendrocytes treated with baclofen or muscimol before exposure to AMPA, and analyzed 10 min or 1 h after AMPA stimulus. All conditions were normalized to values from untreated cells, which was considered as 100% of phosphorylated protein expression levels. Data are shown as the mean ± SEM of at least 3 independent experiments. *p<0.05, **p<0.01, ***p<0.001 vs. control Ø AMPA; #p<0.05, ##p<0.01, ###p<0.001 vs. ØAMPA one‐way ANOVA.

Lastly, we determined whether the protective effects of baclofen or muscimol affected the activation of key molecules in several AMPA-driven signaling pathways in oligodendrocytes. We checked the phosphorylation levels of Src protein kinase, Akt, JNK and CREB in the presence or absence of baclofen or muscimol 30 min before AMPA, and analyzed 10 min or 1 h after AMPA stimulus (Figure 4). Immunoblot analysis proved that AMPA treatment strongly diminished phosphorylated Src (pSrc; Figure 4A) and Akt levels (pAkt; Figure 4B) assessed at a shorter time point (10 min after AMPA stimulus). However, pretreatment with GABAergic agonist did not modify either the basal expression in the absence of AMPA or the changes observed after toxic insult. Similarly, we showed that pJNK (Figure 4C) and pCREB expression (Figure 4D) were increased following AMPA addition (analyzed 1 h after); but, GABAergic drugs did not modulate this activation (Figure 4B). Overall, our results indicate that baclofen and muscimol are not able to restore the changes provoked by AMPA in the tested molecular signaling pathways under our analytical conditions.

## Discussion

Glial cells constitute the vast majority of CNS cells. As described above, oligodendrocytes and myelin are vulnerable to enhanced glutamate signals, and glutamate-induced glial cell death is highly relevant for the pathophysiology of CNS diseases (Stys, 2004; Matute et al., 2006; Micu et al., 2006; Matute et al., 2007; Matute and Ransom, 2012; Fern and Matute, 2019). In humans, white matter (WM) constitutes about half of the CNS volume, which is a greater proportion than in other mammals, including those typically used for animal experiments. This feature may have misrepresented the importance of WM damage for the outcome of CNS diseases such as multiple sclerosis in humans, (Matute, 2011). Mature oligodendrocytes ensheathe axons, potential sources of high glutamate levels, and, they are therefore vulnerable to excitotoxicity. For that reason, the search for drugs that protect oligodendrocytes from this damage will provide novel agents to treat WM injury in the CNS. GABA_B_ and GABA_A_R agonists have been described as displaying neuroprotective roles (Tu et al., 2010; Wei et al., 2012; Hleihil et al., 2021), and recent reports have outlined their importance in oligodendrocyte functionality, proliferation, differentiation and remyelination (Zonouzi et al., 2015; Kalakh and Mouihate, 2019; Serrano-Regal et a., 2020a; Serrano-Regal et al., 2020b; Bai et al., 2021; Serrano-Regal et al., 2022). Here, we demonstrate that baclofen and muscimol, GABA_B_ and GABA_A_R agonists, respectively, exert a protective role in the oligodendrocyte response to glutamate-induced excitotoxicity, by alleviating the damage caused by this insult enhancing the presence of calcium-impermeable GluR2 subunits on the oligodendroglial cell surface.

Previous studies have established that cell-surface expression of GluR2 subunits in OPCs could be reduced by AMPA treatment (Hossain et al., 2014; Harlow et al., 2015), and similarly, we observed that moderate overactivation of AMPAR in mature oligodendrocytes downregulated the expression of GluR2 subunits in the cell membrane. This event is a crucial step in the onset of the excitotoxic program, as the calcium conductance of AMPARs differs markedly depending on whether the GluR2 subunit is a component of the membrane receptor (Hollmann et al., 1991; Jayakar and Dikshit, 2004). Calcium-permeable receptor channels are formed by GluR1, GluR3 or GluR4 subunits, whereas GluR2 subunits restrict calcium entry by rendering the receptor impermeable to calcium. In oligodendrocytes, calcium permeability of AMPARs was shown to be inversely correlated with the abundance of GluR2 subunits on the surface (Deng et al., 2006), indicating that GluR2 is critical for controlling oligodendroglial excitotoxicity.

In this work, we found that the sustained activation of GABARs with baclofen or muscimol stabilized calcium-impermeable GluR2 subunits on the surface of oligodendrocytes. We hypothesize that this stabilization mediates the protective role of baclofen and muscimol, which could prevent the consequent mitochondrial dysfunction through a reduction in calcium influx, the activation of calpain and oligodendrocyte death induced by AMPA. GABAR activation by its agonists may lead to an increase in GABAergic activity that must be neutralized by a decrease in glutamatergic activity, which in this case is achieved by stabilization of GluR2 in the cell membrane and consequent decrease in calcium influx. These compensatory mechanisms could achieve equilibrium in neurotransmitter receptor expression to stabilize the response to synaptic activity. Thus, our results demonstrate that GABAergic drugs alter GluR2 trafficking mechanisms induced by AMPA, most likely through downregulation of endocytosis and/or endosomal recycling of GluR2 subunits. It is also possible that GABAR activation provokes intracellular signals that strengthen interactions between GluR2 subunits and cytoskeletal proteins, providing a more powerful anchoring of the receptor to the membrane. A clear interplay between GABA and glutamate receptors has been previously indicated in neurons, where treatment with baclofen was shown to affect the expression, activity and signaling of glutamate receptors under physiological and pathological conditions, restoring excitatory/inhibitory imbalance (Kantamneni, 2015). However, this close relationship between GABA and GluRs expressed in oligodendrocytes had not been described so far, and a thorough assessment of the molecules and pathways involved in this connection will be necessary, as well as determining its relevance in pathological conditions.

Focusing on the intracellular signaling triggered by the activation of AMPARs in oligodendrocytes, it is well accepted that the increase in cytoplasmic calcium is the key event (Sánchez-Gómez and Matute, 1999), and both baclofen and muscimol reduce the AMPA-induced calcium influx in oligodendrocytes. This reduction in intracellular calcium was observed in combination with a decrease in alterations of mitochondrial membrane potential, ROS production, and calpain activity all promoted by AMPA. ROS formation is closely related to alterations in the mitochondrial membrane potential, and leads to damages in DNA, proteins and lipids, resulting in progressive harmful events leading to cell dysfunction (Suski et al., 2018). Thus, the reduction in mitochondrial membrane potential alteration and consequent reduction in the levels of ROS caused by GABAergic drugs reinforces the role of baclofen and muscimol on cell survival and protection against excitotoxicity. Calpain activation is also known to induce cell dysfunction by proteolytic cleavage of various targets involved in apoptotic or necrotic pathways in addition to causing direct alterations in mitochondria (Mansouri et al., 2006; Sánchez-Gómez et al., 2011). Among the proteins targeted by calpain, we find Src protein kinase, which is cleaved by these proteases generating a truncated fragment that induces cell death in neurons and is related to inactivation of Akt signaling (Hossain et al., 2013). Src and Akt are mediators of key survival signaling pathways, and our results showed their early inactivation after AMPA stimulus (10 min). Moreover, we have previously described that AMPA triggered JNK and CREB activation (Canedo-Antelo et al., 2018); other authors have also shown the involvement of calpains in the processing of these molecules (Trinchese et al., 2008; Bollino et al., 2015; Messaoud et al., 2015). However, baclofen and muscimol did not seem to modulate these signaling mechanisms, at least during the times analyzed, despite reducing AMPA-induced calpain activity. Therefore, the molecules and/or pathways involved in the protective effect of baclofen or muscimol from excitotoxic damage caused by AMPA have not yet been determined. Further investigations are necessary to clarify the molecules and pathways involved in the oligoprotection exerted by baclofen or muscimol. The signaling axis involving Smac/DIABLO or p38 could be a promising target, given its relevance in calpain-targeted activity (Bollino et al., 2015; Messaoud et al., 2015).

GluR2 overexpression in OPCs favors not only OPC proliferation, but also oligodendrocyte regeneration following demyelinating brain injury, which suggests that suppressing AMPAR calcium signaling in OPCs could help to promote myelin repair (Khawaja et al., 2021). In this context, the restoration of cell-surface levels of GluR2 subunits in oligodendrocytes after AMPA insult, induced by baclofen and muscimol, could be relevant for the regenerative response after CNS damage and we endorse these drugs as promising tools for limiting oligodendrocyte death and myelin damage in demyelinating diseases.

Interestingly, we have observed that the muscimol effect is more homogeneous and robust than baclofen. Muscimol was able to reduce AMPA-induced cell death when applied after AMPA stimulus, increase GluR2 cell-surface expression even in the absence of AMPA, and reduce AMPA-associated calcium uptake when added 2-3 DIV before the recordings. This more consistent effect, independent of the time or duration of drug treatment, may be related to the faster response that ionotropic GABA_A_R activation causes in ion trafficking, compared to the metabotropic GABA_B_R (Frangaj and Fan, 2018). Nevertheless, given the positive neuroprotective effect resulting from co-activation of GABA receptors using muscimol and baclofen (Wei et al., 2012), a combination of GABAR agonists baclofen and muscimol may lead to a synergistic effect more powerful than the one exerted by each drug alone.

In conclusion, the present study shows how GABAR agonists, baclofen and muscimol, affect AMPA-induced excitotoxicity in oligodendrocytes by modulating calcium-related signaling and reducing cell death. Further analyses will clarify the exact mechanism of action of baclofen and muscimol to disrupt AMPA-induced damaging effect in oligodendrocytes and will confirm the observed protective effect of these drugs using in vivo models of CNS diseases related to excitotoxicity, including brain ischemia, or demyelinating diseases such as multiple sclerosis.

## Data Availability

The raw data supporting the conclusions of this article will be made available by the authors, without undue reservation.
